# Improved Resting-State Functional Dynamics in Post-stroke Depressive Patients After Shugan Jieyu Capsule Treatment

**DOI:** 10.3389/fnins.2020.00297

**Published:** 2020-04-16

**Authors:** Guanqun Yao, Jing Li, Jiaojian Wang, Sha Liu, Xinrong Li, Xiaohua Cao, Huafu Chen, Yong Xu

**Affiliations:** ^1^Department of Psychiatry, First Hospital/First Clinical Medical College of Shanxi Medical University, Taiyuan, China; ^2^Shanxi Key Laboratory of Artificial Intelligence Assisted Diagnosis and Treatment for Mental Disorder, First Hospital of Shanxi Medical University, Taiyuan, China; ^3^The Clinical Hospital of Chengdu Brain Science Institute, MOE Key Lab for Neuroinformation, School of Life Sciences and Technology, University of Electronic Science and Technology of China, Chengdu, China; ^4^MDT Center for Cognitive Impairment and Sleep Disorders, First Hospital of Shanxi Medical University, Taiyuan, China

**Keywords:** post-stroke depression, Shugan Jieyu Capsule, cognitive function, functional magnetic resonance imaging, dynamic amplitude of low-frequency fluctuations, dynamic functional connectivity

## Abstract

Shugan Jieyu Capsule (SG), a Chinese herbal medicine mainly composed of *Acanthopanax* and *Hypericum perforatum*, has been used to ameliorate cognitive impairments and emotional problems induced by post-stroke depression (PSD), while the altered brain dynamics underlying the ameliorative effects of SG have remained unclear. Our study focused on investigating the potential neurobiological mechanisms of SG in improving the cognitive function of PSD patients via resting-state functional magnetic resonance imaging (fMRI). Fifteen PSD patients (mean ages: 64.13 ± 6.01 years) were instructed to take 0.72 g of SG twice a day for 8 weeks. PSD patients underwent fMRIs, the 24-item Hamilton Depression Scale (HAMD-24) and the Montreal Cognitive Assessment (MoCA) at baseline and the end of intervention, and these assessments were also performed on twenty-one healthy controls (HC) (mean ages: 60.67 ± 6.95 years). Additionally, the dynamic amplitude of low-frequency fluctuations (dALFF) and functional connectivity (dFC) were determined to reveal changes in dynamic functional patterns. We found that taking SG significantly reduced the depressive symptoms assessed by HAMD-24 and improved cognitive functions assessed by MoCA in PSD patients. Furthermore, at baseline, PSD patients showed decreased dALFF in the right precuneus and increased dFC between the right precuneus and left angular gyrus, compared with HC. After intervention, the dALFF and dFC variances of the abnormal patterns were reversed. Additionally, the dALFF variance in the right precuneus was positively correlated with MoCA scores in PSD patients after SG treatment. Collectively, our results indicate that SG may improve the cognitive function of PSD patients through alteration of brain dynamics. Our findings lay a foundation for the exploration of the neurobiological mechanisms of SG in ameliorating symptoms of PSD patients.

## Introduction

Post-stroke depression (PSD), a disease that leads to permanent tissue damage caused by thrombotic and/or hemorrhagic events, refers to psychiatric symptoms such as sleeplessness, depressed mood, and inferiority (Bai et al., 2017). Individuals with PSD exhibit poor rehabilitation outcomes, social isolation, suicidal ideation, and suicide attempts, all of which affect recovery, prolong hospital stays and increase the economic burden compared to non-depressed stroke patients (Cai et al., 2019). With the physiological defect and social dysfunction, PSD also causes cognitive impairments such as attentional decline, impaired memory, and impaired motor function (Quaranta et al., 2012).

Some theories have been proposed to account for the mechanisms of PSD. One theory is that a large vessel occlusion may result in ischemic necrosis to deteriorate cortical functions (Wang et al., 2009), while another theory posits that neural circuits involved in cognition are abnormal because of aberrant production and transmission of neurotransmitters after stroke (Gabrieli, 1996). Furthermore, amyloid deposits on white matter fibers caused by brain damage may also contribute to cognitive decline in PSD patients (Gold et al., 2005). Recent studies have demonstrated that PSD is not related to the locations of lesions, whereas PSD has been shown to be associated with dysfunction in specific brain networks such as the salience network and default mode network (Loubinoux et al., 2012). Therefore, further elucidation of functional brain abnormalities in PSD patients may help to guide the development of more effective treatments.

Shugan Jieyu Capsule (SG) is a Chinese herbal medicine that is primarily made up of *Acanthopanax* and *Hypericum perforatum* (Yang, 2015) and has been licensed and widely prescribed for depression in China since 2008 (Sun et al., 2009). *Acanthopanax*, commonly known as ciwujia or Siberian ginseng, is a traditional Chinese medicine that ameliorates cognitive dysfunction induced by cholinergic blockade (Li et al., 2016). *Hypericum perforatum*, also called St. John’s wort, has been used as an antidepressant agent and a cognitive enhancer in rodents (Avila et al., 2018). *Hypericum perforatum* has been shown to induce positive effects on both mood and short-term verbal memory (Yechiam et al., 2019). The effects of SG are similar to those of escitalopram in terms of ameliorating depressive symptoms, while SG causes fewer adverse reactions (Sun et al., 2018; Huang and Kuang, 2019). Furthermore, SG has been used to ameliorate depression, and improve cognitive function in PSD patients (Zhen et al., 2018), as well as to increase their levels of norepinephrine and serotonin and to inhibit inflammation (Liu et al., 2019). However, potential neurological mechanisms behind SG’s improvement of the cognition of PSD patients have not been proposed.

Functional magnetic resonance imaging (fMRI), a non-invasive measurement for indirectly mapping brain function via hemodynamic changes (Maguire, 2012), has been utilized to study the neurobiological mechanisms underlying the effects of traditional Chinese medicines. Additionally, some studies have found that impaired cognitive function is related to altered functions in specific regions – such as the precuneus, anterior cingulate cortex, and medial precentral gyrus (Bruner et al., 2017) – and those depressive symptoms are related to aberrant functioning in some brain networks, such as the fronto-parietal network and default mode network (DMN) (Korgaonkar et al., 2019). Recently, dynamic functional activity and connectivity analyses have been used to measure temporal flexibility in spontaneous fluctuations (Thompson, 2018). The major advantage of dynamics over static measures is the ability to capture recurring brain activity (Xie et al., 2018), and specific exploration of dynamics may complement deficiencies in static alterations. This method is repeatable at different times and in diverse participants, and it has shown excellent stability (Shen et al., 2017), which suggests that dynamic properties may reveal neuropathological changes in PSD and guide related treatments.

The amplitude of low-frequency fluctuations (ALFF) reflects the activity of spontaneous neurons (Hoptman et al., 2010) and is used to locate abnormal functional activities associated with various diseases by calculating the amplitude of low-frequency oscillation of each voxel. Functional connectivity (FC) is characterized by the synchronization of activities in functional brain regions and is often used to reflect direct or indirect connections between brain regions (Marrelec et al., 2016). Furthermore, the dynamic ALFF (dALFF) based on time-variant brain activity emphasize the abnormal functional activity in psychological and cognitive disorders (Cui et al., 2019). Dynamic functional connectivity (dFC), an emerging functional connectivity technology, can be used to measure the strength of functional connectivity and the variability of spatial dynamic organization (Cohen, 2018). The dynamical neuroimaging methods, including dALFF and dFC, have been shown to be effective for elucidating the temporal variability of neural circuits across sensorimotor, emotion, attention, and memory functions (Liao et al., 2014). Additionally, these dynamics have been used to explore the mechanism of drugs acting on diseases. For example, a study demonstrated that risperidone treatment could reverse the abnormal temporal variability of insular subdivisions, and the changes in dFC were correlated with the reduction of Positive and Negative Symptom Scale scores (Duan et al., 2019). These findings suggest that dALFF and dFC are effective tools for assessing cognitive function and investigating the neurobiological mechanisms of traditional herbal Chinese medicines.

Based on previous evidence, we hypothesized that administrating SG may ameliorate the cognitive dysfunction of PSD patients and that this improvement would be related to changes in brain dynamics. To test this hypothesis, we employed the 24-item Hamilton Depression Scale (HAMD-24) to assess depression symptoms and the Montreal Cognitive Assessment Scale (MoCA) (Gagnon et al., 2010) to assess cognition, and we used dALFF and dFC to investigate the potential neurobiological mechanisms of SG.

## Materials and Methods

### Participants

Fifteen right-handed PSD patients (eight males; age range: 50–70 years, mean ages: 64.13 ± 6.01 years) were enrolled in the SG group from the psychiatry department of three Taiyuan hospitals, and 21 age-, sex-, handedness-, and educational level-matched healthy volunteers (HC) were recruited. Our research was authorized by the Ethics Committee of the Shanxi Medical University (Shanxi, China), and each participant signed an informed consent form. This study was registered in the Chinese Clinical Trial Registry (registration number: ChiCTR1900026358). All patients met the following inclusion criteria: (1) first hemorrhagic or ischemic stroke diagnosed by CT or MRI; (2) depression occurring shortly after the occurrence of a stroke (within 1 week to 3 months); (3) mild to moderate depression, as indicated by a HAMD-24 between 8 and 24; (4) age between 50 and 70 years, and confirmed to be right-handed and of Han ethnicity; and (5) clear consciousness, with no dementia, aphasia, or other symptoms, and able to complete psychological tests and other tests. The following exclusion criteria were applied: (1) claustrophobia, heart stents, metal dentures, or other physical limitations prohibiting MRI examination; (2) other severe organic diseases (e.g., severe heart, lung, liver, or kidney dysfunction, or severe infection); (3) taking antidepressants or other drugs that affect mental activity, or having received electroconvulsive therapy before participating in the present study; (4) presence of other mental disorders or substance abuse; and (5) severe depression (HAMD-24 > 24).

### Study Design

**FIGURE 1:**
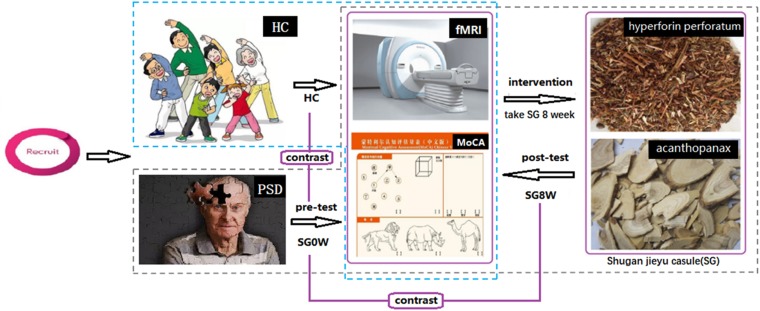
Study flow-chart. Abbreviations are as follows: HC, healthy controls; PSD, post-stroke depression; SG0W, PSD patients before SG treatment; SG8W, PSD patients after 8 weeks of SG treatment; fMRI, functional magnetic resonance imaging; MoCA, Montreal Cognitive Assessment Scale; SG, Shugan Jieyu Capsule.

In this study, all patients were diagnosed with depressive symptoms within 3 days. All participants completed resting-state fMRI scans and were assessed with HAMD-24 and MoCA at baseline. After pre-tests, PSD patients were instructed to take 1.44 g of SG a day (0.72 g in the morning and 0.72 g in the evening) for 8 weeks, which was the recommended dose in the medicine instructions. After 8 weeks, fMRI scans, HAMD-24 and MoCA of the PSD group were acquired again, without a washout period. The study flow-chart is shown in Figure 1.

### Data Acquisition

HAMD-24 and MoCA were performed to assess depressive symptoms and cognitive function. HAMD-24, which was developed by Hamilton in 1960, is the most common assessment tool for depressive symptoms (Yan et al., 2016). MoCA has high sensitivity and specificity and contains eight cognitive domains: visuospatial and executive function, denomination, memory, attention, language, abstract thinking, delayed memory and orientation (Gagnon et al., 2010).

All MRI data were obtained by a 3.0 Tesla Siemens MRI whole-body scanner at Shanxi Provincial People’s Hospital, Shanxi, China. Headsets and foam pads were employed to minimize noise and head motion. The participants were required to shut their eyes quietly, keep their minds clear, and not think deeply about anything. The 3D T1 imaging was acquired as follows: repetition time/echo time (TR/TE) = 2,300/2.95 ms; matrix = 240 × 256; flip angle = 9°; voxel size = 0.9 mm × 0.9 mm × 1.2 mm; and 160 transverse slices without a slice gap. Functional images had the following parameters: 212 whole-brain volumes for each participant; 32 transverse slices without a slice gap; repetition time/echo time = 2,500/30 ms; flip angle = 90°; voxel size = 3.75 mm × 3.75 mm × 4 mm.

### Lesion Mapping

**FIGURE 2:**
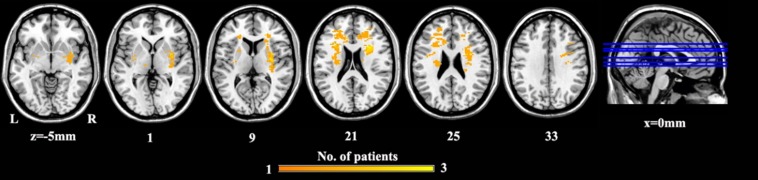
The lesion map for PSD patients. Voxels that are damaged in at least one patient are projected on the standard template in MNI space. Abbreviations are as follows: MNI, Montreal Neurological Institute space; PSD, post-stroke depression.

A lesion overlap map of PSD patients was constructed (Figure 2). A neurosurgeon (W.X.) carefully marked the location of the lesion on individual 3D T1 images. A lesion mask for each PSD patient was created using MRIcron^1^. After the spatial normalization, the combination of lesion masks was created to construct a group lesion map for all the participants.

### Imaging Preprocessing

Resting-state functional data were obtained using SPM12^2^ and DPARSF^3^ toolkits. We discarded the first 10 time points to adapt to the participants’ signal equilibrium and scanning noise. By comparing all the functional images to the first image, we corrected for the head motion. No translational or rotational parameters exceeding ± 2.5 mm or ± 2.5°, were found in any subject. The 3D T1-based transformation was used to perform the spatial normalization of the functional images. The 3D T1 images were co-registered to mean functional images. We segmented and normalized the 3D T1 images to the Montreal Neurologic Institute (MNI) space through a 12-parameter non-linear transformation. Furthermore, to avoid differences in the spatial normalization, a cost-function modification was performed to exclude the lesion area (Brett et al., 2001). These transformation parameters were applied to the functional imaging. After spatial normalization, functional data were further re-sampled to a voxel size of 2 mm × 2 mm × 2 mm. Afterward, functional imaging was spatially smoothed with a Gaussian kernel of full-width at half-maximum of 6 mm. Furthermore, the linear trends were removed from the time series, and temporal band-pass filtering (0.01–0.08 Hz) was applied to functional signals. In addition, to reduce the influence of non-neuronal fluctuations and head motion, white matter signals, 24 motion parameters, and cerebrospinal fluid were discarded as nuisance variables.

### dALFF Analysis

Dynamic ALFF was calculated via the DynamicBC toolbox characterized by a sliding-window method (Liao et al., 2014). We set the sliding window size to 50 TRs (125 s) and the sliding step size to 5 TRs (10 s). Each subject was divided into 31 windows, and the ALFF map was acquired within each sliding window. We utilized additional window lengths [30 TRs (75 s) and 80 TRs (200 s)] to verify our dALFF findings. Additionally, to investigate the temporal variability of brain activity, we calculated the variance of the ALFF map of each subject under all windows. Finally, the Fisher-z transformation was used for converting the dALFF variance of each voxel to a z-score to enhance data normality.

### dFC Analysis

In addition, brain regions with significant differences in the dALFF analysis were used as seed regions, and inter-regional dFC was analyzed. We acquired the dFC maps of each seed region via the DynamicBC toolbox. The window lengths were consistent with the dALFF analysis. Pearson’s correlation coefficients between the mean signals of each region and the signals of every other voxel were computed. Furthermore, we calculated the variance of the FC map of each subject under all windows. Finally, the Fisher z-transformation was used to convert the dFC variance to a z-score to enhance data normality.

### Data Analysis

We utilized paired-sample *t*-tests using SPSS 22.0 software to compare the scale scores of PSD patients at baseline (SG0W) and at the end of the intervention (SG8W). The differences in scale scores between SG-treated patients and HC were compared with two-sample *t*-tests using SPSS 22.0. FDR correction at *p* < 0.05 was performed to correct the comparisons of the scale scores. A paired-sample *t*-test was used to assess the differences in dALFF variance between SG0W and SG8W. Corrections for multiple comparisons were determined by Gaussian random field (GRF)-corrected voxel *p*-values <0.01, and cluster *p*-values <0.05. The same steps were taken to perform between-group comparisons of dFC.

The mean dALFF and dFC variances of the clusters, which showed significant differences between SG0W and SG8W, were extracted to conduct a *post hoc* comparison. Corrections for multiple comparisons were determined by FDR-correction with *p* < 0.05. Pearson’s correlation analysis was performed to identify whether the associations of the altered dALFF and dFC variances were correlated with cognitive function (MoCA) and/or depression symptoms (HAMD-24) before and after SG treatment.

## Results

### Demographics

**FIGURE 3:**
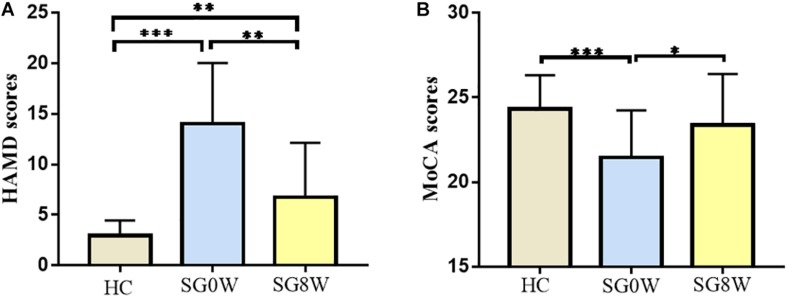
Clinical characteristics of participants. The histograms show HAMD **(A)** and MoCA **(B)** scores for each group (FDR correction at *p* < 0.05). ^∗^*p* < 0.05, ^∗∗^*p* < 0.01, ^∗∗∗^*p* < 0.001. **(A)** HAMD: HC vs. SG0W (*t* = 8.103, *p* < 0.001), HC vs. SG8W (*t* = 3.061, *p* = 0.004), SG0W vs. SG8W (*t* = 3.911, *p* = 0.002). **(B)** MoCA: HC vs. SG0W (*t* = 3.622, *p* < 0.001), HC vs. SG8W (*t* = 1.126, *p* = 0.268), SG0W vs. SG8W (*t* = 2.327, *p* = 0.035). The results are mean ± standard deviation. Abbreviations are as follows: MoCA, Montreal Cognitive Assessment Scale; HAMD, Hamilton Depression Scale; SG0W, PSD patients before SG treatment; SG8W, PSD patients after 8 weeks of SG treatment; HC, healthy controls; PSD, post-stroke depression; SG, Shugan Jieyu Capsule.

The demographics of the subjects are displayed in Table 1. No differences in gender (χ^2^ = 0.3853, *p* = 0.535), age (Mann–Whitney *U* test, *p* = 0.181), or level of education (Mann–Whitney *U* test, *p* = 0.241) were observed. The mean lesion volume of PSD patients was 1.65 ± 1.46 cm^3^. Compared to HC, the scale scores of MoCA in SG0W were decreased (*p* < 0.05, FDR correction), whereas the scores in SG8W did not show a group difference. Compared to those of HC, the scale scores of HAMD-24 in SG0W were increased (*p* < 0.05, FDR correction). Additionally, compared to those of SG0W, the scale scores of HAMD in SG8W were significantly decreased (*p* < 0.05, FDR correction). The scale scores of MoCA and HAMD in SG0W, SG8W, and HC are shown in Figure 3.

**TABLE 1:** Demographics.

	SG0W	SG8W	HC		
	(*n* = 15)	(*n* = 15)	(*n* = 21)		
Variable	Mean ± SD	Mean ± SD	Mean ± SD	*t/χ^2^*	*p*
Age (years)	64.13 ± 6.01		60.67 ± 6.95	–	0.181^a^
Gender (male/female)	8/7		9/12	0.385	0.535^b^
Education (years)	9.40 ± 3.38		10.29 ± 2.61	–	0.241^a^
Duration (days)	62.87 ± 15.73				
**Stroke type**					
Ischemia	9(60.0)				
Hemorrhage	6 (40.0)				
Lesion volume (cm^3^)	1.65 ± 1.46				
**Location of lesion**					
Basal ganglia	9(60.0)				
Frontal lobe	3(20.0)				
Thalamus	3(20.0)				

*SG0W, post-stroke depressive patients before SG treatment; SG8W, post-stroke depressive patients after 8 weeks of SG treatment; HC, healthy controls; Duration, the time of stroke from diagnosis to recruitment; a, Mann-Whitney *U*-tests; b, chi-squared tests; SG, Shugan Jieyu Capsule.*

### Differences in dALFF

**FIGURE 4:**
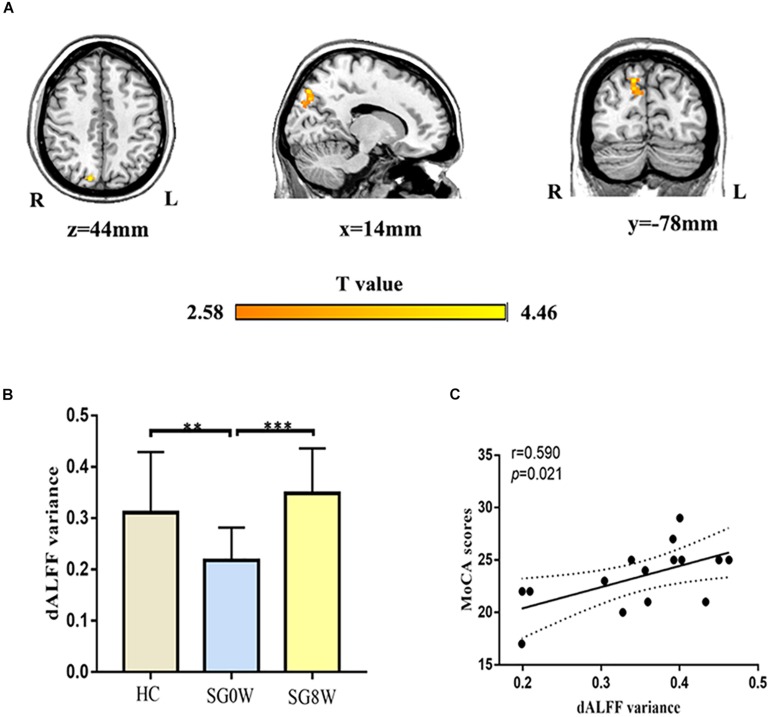
Differences in dALFF and correlation between dALFF and MoCA. **(A)** Group differences in the altered dALFF variance for the right precuneus (MNI coordinates, *x* = 14, *y* = −78, *z* = 44, cluster size = 111 voxels, *t* = 4.460, *p* < 0.05 GRF correction). **(B)** The histogram shows the average dALFF variance in the right precuneus (FDR correction at *p* < 0.05). ^∗∗^*p* < 0.01, ^∗∗∗^*p* < 0.001. HC vs. SG0W (Mann-Whitney *U* test, *p* = 0.008), HC vs. SG8W (Mann-Whitney *U* test, *p* = 0.102), SG0W vs. SG8W (*t* = 6.616, *p* < 0.001). These results are mean ± standard deviation. **(C)** This correlation diagram shows the correlation between MoCA and average dALFF variance for the right precuneus in SG8W (*r* = 0.590, *p* = 0.021). The solid line and dashed line represent the best-fit line and 95% confidence interval of Pearson’s correlation, respectively. Abbreviations are as follows: L, left; R, right; dALFF, dynamic amplitude of low-frequency fluctuation; MoCA, Montreal Cognitive Assessment Scale; SG0W, PSD patients before SG treatment; SG8W, PSD patients after 8 weeks of SG treatment; HC, healthy controls; PSD, post-stroke depression; SG, Shugan Jieyu Capsule.

We mainly utilized the 50 TRs (125 s) window length to conduct the dALFF analysis. SG8W exhibited increased dALFF in the right precuneus (MNI coordinates *x* = 14, *y* = −78, *z* = 44, cluster size = 111 voxels, *t* = 4.460, *p* < 0.05 GRF correction) compared to SG0W (Figure 4). The analyses of additional window lengths (75 and 200 s) are displayed in Supplementary Figure 1.

### Differences in dFC

**FIGURE 5:**
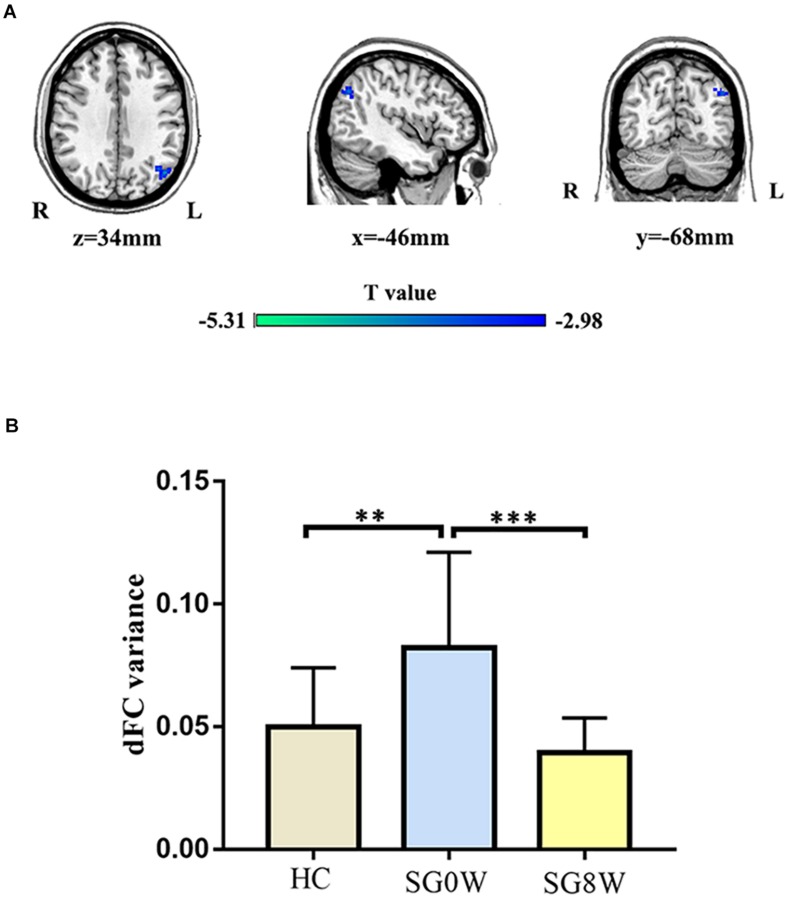
Differences in dFC. **(A)** Group differences in dFC variance for the right precuneus and left angular gyrus (MNI coordinates: *x* = −46, *y* = −68, *z* = 34, cluster size = 135 voxels, *t* = −4.270, *p* < 0.05 GRF correction). **(B)** The histogram shows the mean dFC variance between the right precuneus and left angular gyrus (FDR correction at *p* < 0.05). These results are mean ± standard deviation. ^∗∗^*p* < 0.01, ^∗∗∗^*p* < 0.001. HC vs. SG0W (Mann-Whitney *U* test, *p* = 0.002), HC vs. SG8W (Mann-Whitney *U* test, *p* = 0.505), SG0W vs. SG8W (*t* = 4.622, *p* < 0.001). Abbreviations are as follows: L, left; R, right; dFC, dynamic functional connectivity; SG0W, PSD patients before SG treatment; SG8W, PSD patients after 8 weeks of SG treatment; HC, healthy controls; PSD, post-stroke depression; SG, Shugan Jieyu Capsule.

The seed regions of dFC were mainly based on the brain areas with significantly changed dALFF. For the right precuneus, SG8W showed decreased dFC between the right precuneus and left angular gyrus (MNI coordinates: *x* = −46, *y* = −68, *z* = 34, cluster size = 135 voxels, *t* = −4.270, *p* < 0.05 GRF correction) (Figure 5).

### Correlation Analysis

As shown in Figure 4, there was a significant correlation between the dALFF variance in the right precuneus and the MoCA scores (*r* = 0.590, *p* = 0.021) in SG8W.

## Discussion

In this study, we unitized MoCA to assess the cognitive functions of PSD patients. We found that the cognitive function of PSD patients was lower than that of HC and that taking SG improved their cognitive function, which was similar to previous results reported for patients with cognitive impairment (Ma, 2016). We also found that SG significantly increased the dALFF in the right precuneus and decreased the dFC between the right precuneus and left angular gyrus in PSD patients. Collectively, our results suggest that SG may enhance cognitive function in PSD patients by changing the brain dynamics of resting-state functional activity and functional connectivity.

Both the precuneus and the angular gyrus are important regions of the DMN, which is one of the key brain networks related to self-projection and memory (Wang et al., 2018). The DMN circuit is more active at rest than during tasks in most cases and is also active when participants are engaged in involuntary conscious activity such as fantasy, understanding, and distraction (Kaulmann et al., 2017). Some studies have shown that the DMN is crucial to the mental symptoms of PSD patients, especially depression and irritability (Liu et al., 2014). Furthermore, hyper-functioning of the DMN in major depressive disorder has been related to lower adaptive self-reflection and higher maladaptive rumination (Wang et al., 2019). We speculate that SG may change the abnormal brain dynamics of PSD patients in the DMN. Furthermore, the right precuneus is a part of the posterior parietal cortex (PPC), which is primarily a region implicated in cognitive function, especially in regulating sensation and movement, and can rapidly acquire a memory representation during learning (Kaulmann et al., 2017). Additionally, the PPC is a central interface that integrates visual, cognitive, and motor-related signals. The PPC exhibits the ability to store memory, especially in later memory recall after a prolonged standby state (Wong and Lomber, 2019).

In terms of brain regions, studies have shown that the precuneus and the angular gyrus also play important roles in cognitive function. The precuneus is associated with many high-level cognitive functions, such as self-processing and consciousness (Li et al., 2018). There is evidence that the precuneus may be related to the integration of various neural circuits to generate a conscious self-perception (Amico et al., 2017). The left angular gyrus, an intermediary variable factor between perception and interpretation, is involved in coding subjective memory (Molinaro et al., 2015). Additionally, studies have shown that the DMN, precuneus, and angular gyrus are significantly correlated with MoCA scores (Possin et al., 2018). Our results were basically consistent with the previous studies, demonstrating that the altered activity of the right precuneus was correlated with MoCA scores and that SG may improve the cognitive function of PSD patients by changing the abnormal dynamics of the DMN and PPC. A comparison of SG8W with SG0W revealed a reversal of dALFF variance in the right precuneus, suggesting that SG administration had an effective action on the dynamics of brain activity and that the right precuneus might be a promising target for SG treatment.

However, there are some limitations to our research. First, the number of subjects was small. Therefore, larger sample sizes are needed for future studies. Second, the control group that we used may not be ideal. PSD patients subjected to a placebo treatment were not used as the control group due to time and funding limitations. Third, our study did not assess the long-term outcomes of participants. In conclusion, our study demonstrated that taking SG improves the cognitive function of PSD patients and is correlated with SG-induced changes in the dynamics of intrinsic brain activity. These results lay a foundation for the exploration of the neurobiological mechanisms of SG in ameliorating symptoms of PSD patients.

## Data Availability Statement

The datasets generated for this study are available on request to the corresponding author.

## Ethics Statement

The studies involving human participants were reviewed and approved by the Ethics Committee of the Shanxi Medical University (Shanxi, China). The patients/participants provided their written informed consent to participate in this study.

## Author Contributions

YX and HC designed and supervised the study. GY focused on analysis of experimental data and accomplished the essay. JL and XL helped to operate the experimental procedures and revised the manuscript. SL, JW, and XC contributed to recruiting participants, reviewing the data analyses, and revising the literature.

## Conflict of Interest

The authors declare that the research was conducted in the absence of any commercial or financial relationships that could be construed as a potential conflict of interest.

## Footnotes

1 http://www.mccauslandcenter.sc.edu/mricro/mricron/

2 http://www.fil.ion.ucl.ac.uk/spm

3 http://www.restfmri.net

## References

[B1] Amico E. Marinazzo D. Di Perri C. Heine L. Annen J. Martial C. (2017). Mapping the functional connectome traits of levels of consciousness. *Neuroimage* 148 201–211. 10.1016/j.neuroimage.2017.01.020 28093358

[B2] Avila C. Whitten D. Evans S. (2018). The safety of St John’s wort (*Hypericum perforatum*) in pregnancy and lactation: a systematic review of rodent studies. *Phytother. Res.* 32 1488–1500. 10.1002/ptr.6099 29708295

[B3] Bai Y. Wang Y. L. Shantsila A. Lip G. Y. H. (2017). The global burden of atrial fibrillation and stroke: a systematic review of the clinical epidemiology of atrial fibrillation in Asia. *Chest* 152 810–820. 10.1016/j.chest.2017.03.048 28427968

[B4] Brett M. Leff A. P. Rorden C. Ashburner J. (2001). Spatial normalization of brain images with focal lesions using cost function masking. *Neuroimage* 14 486–500. 10.1006/nimg.2001.0845 11467921

[B5] Bruner E. Preuss T. M. Chen X. Rilling J. K. (2017). Evidence for expansion of the precuneus in human evolution. *Brain Struct. Funct.* 222 1053–1060. 10.1007/s00429-015-1172-y 26725108 PMC4930733

[B6] Cai W. Mueller C. Li Y. J. Shen W. D. Stewart R. (2019). Post stroke depression and risk of stroke recurrence and mortality: a systematic review and meta-analysis. *Ageing Res. Rev.* 50 102–109. 10.1016/j.arr.2019.01.013 30711712

[B7] Cohen J. R. (2018). The behavioral and cognitive relevance of time-varying, dynamic changes in functional connectivity. *Neuroimage* 180(Pt B) 515–525. 10.1016/j.neuroimage.2017.09.036 28942061 PMC6056319

[B8] Cui Q. Sheng W. Chen Y. Pang Y. Lu F. Tang Q. (2019). Dynamic changes of amplitude of low-frequency fluctuations in patients with generalized anxiety disorder. *Hum. Brain Mapp.* 41 1667–1676. 10.1002/hbm.24902 31849148 PMC7267950

[B9] Duan X. Hu M. Huang X. Su C. Zong X. Dong X. (2019). Effect of risperidone monotherapy on dynamic functional connectivity of insular subdivisions in treatment-naive, first-episode schizophrenia. *Schizophr. Bull.* [Epub ahead of print]. 31504959 10.1093/schbul/sbz087 PMC7147596

[B10] Gabrieli J. D. (1996). Memory systems analyses of mnemonic disorders in aging and age-related diseases. *Proc. Natl. Acad. Sci. U.S.A.* 93 13547–13551. 10.1073/pnas.93.24.13534 8942968 PMC33642

[B11] Gagnon J. Postuma R. Joncas S. Desjardins C. Latreille V. (2010). The montreal cognitive assessment: a screening tool for mild cognitive impairment in REM sleep behavior disorder. *Mov. Dis.* 25 936–940. 10.1002/mds.23079 20310038

[B12] Gold G. Kovari E. Herrmann F. R. Canuto A. Hof P. R. Michel J. P. (2005). Cognitive consequences of thalamic, basal ganglia, and deep white matter lacunes in brain aging and dementia. *Stroke* 36 1184–1188. 10.1161/01.STR.0000166052.89772.b5 15891000

[B13] Hoptman M. J. Zuo X. N. Butler P. D. Javitt D. C. D’Angelo D. Mauro C. J. (2010). Amplitude of low-frequency oscillations in schizophrenia: a resting state fMRI study. *Schizophr. Res.* 117 13–20. 10.1016/j.schres.2009.09.030 19854028 PMC2822110

[B14] Huang Q. Kuang Z. W. (2019). Clinical effect of Shugan Jieyu capsule combined with escitalopram in the treatment of senile depression. *Contemp. Med.* 2019 80–81.

[B15] Kaulmann D. Hermsdorfer J. Johannsen L. (2017). Disruption of right posterior parietal cortex by continuous Theta Burst Stimulation alters the control of body balance in quiet stance. *Eur. J. Neurosci.* 45 671–678. 10.1111/ejn.13522 28092413

[B16] Korgaonkar M. S. Goldstein-Piekarski A. N. Fornito A. Williams L. M. (2019). Intrinsic connectomes are a predictive biomarker of remission in major depressive disorder. *Mol. Psychiatry* 10.1038/s41380-019-0574-2 31695168 PMC7303006

[B17] Li B. Li X. Pan Y. Qiu J. Zhang D. (2018). The relationship between self-enhancing humor and precuneus volume in young healthy individuals with high and low cognitive empathy. *Sci. Rep.* 8:3467. 10.1038/s41598-018-21890-0 29472593 PMC5823885

[B18] Li T. Ferns K. Yan Z. Q. Yin S. Y. Kou J. J. Li D. (2016). *Acanthopanax senticosus*: photochemistry and anticancer potential. *Am. J. Chin. Med.* 44 1543–1558. 10.1142/S0192415X16500865 27852123

[B19] Liao W. Wu G. R. Xu Q. Ji G. J. Zhang Z. Zang Y. F. (2014). DynamicBC: a MATLAB toolbox for dynamic brain connectome analysis. *Brain Connect.* 4 780–790. 10.1089/brain.2014.0253 25083734 PMC4268585

[B20] Liu J. Qin W. Wang H. Zhang J. Xue R. Zhang X. (2014). Altered spontaneous activity in the default-mode network and cognitive decline in chronic subcortical stroke. *J. Neurol. Sci.* 347 193–198. 10.1016/j.jns.2014.08.049 25304057

[B21] Liu S. Q. Zhang L. N. Yuan C. (2019). Clinical efficacy of Shugan jieyu capsule in the treatment of post-stroke depression and its effect on the levels of norepinephrine and 5-hydroxytryptamine. *World Chin. Med.* 14 1784–1788. 10.3969/j.issn.1009-4393.2019.30.033

[B22] Loubinoux I. Kronenberg G. Endres M. Schumann-Bard P. Freret T. Filipkowski R. K. (2012). Post-stroke depression: mechanisms, translation and therapy. *J. Cell Mol. Med.* 16 1961–1969. 10.1111/j.1582-4934.2012.01555.x 22348642 PMC3822966

[B23] Ma W. (2016). The influence of Shugan jieyu capsules on cognitive function and sleep quality in the treatment of depression after stroke. *Chin. Health Stand. Manag.* 7 136–138.

[B24] Maguire E. A. (2012). Studying the freely-behaving brain with fMRI. *Neuroimage* 62 1170–1176. 10.1016/j.neuroimage.2012.01.009 22245643 PMC3480644

[B25] Marrelec G. Messe A. Giron A. Rudrauf D. (2016). Functional connectivity’s degenerate view of brain computation. *PLoS Comput. Biol.* 12:e1005031. 10.1371/journal.pcbi.1005031 27736900 PMC5063374

[B26] Molinaro N. Paz-Alonso P. M. Dunabeitia J. A. Carreiras M. (2015). Combinatorial semantics strengthens angular-anterior temporal coupling. *Cortex* 65 113–127. 10.1016/j.cortex.2015.01.004 25682046

[B27] Possin K. L. Moskowitz T. Erlhoff S. J. Rogers K. M. Johnson E. T. Steele N. Z. R. (2018). The brain health assessment for detecting and diagnosing neurocognitive disorders. *J. Am. Geriatr. Soc.* 66 150–156. 10.1111/jgs.15208 29355911 PMC5889617

[B28] Quaranta D. Marra C. Gainotti G. (2012). Post-stroke depression: main phenomenological clusters and their relationships with clinical measures. *Behav. Neurol.* 25 303–310. 10.1155/2012/501979 22713379 PMC5294256

[B29] Shen R. Junhua L. Taya F. deSouza J. Thakor N. V. Bezerianos A. (2017). Dynamic functional segregation and integration in human brain network during complex tasks. *IEEE Trans. Neural. Syst. Rehabil. Eng.* 25 547–556. 10.1109/TNSRE.2016.2597961 28113670

[B30] Sun X. Chen A. Xu X. Zhang H. Tang Q. Zhang H. (2009). A randomized, double-blind, placebo-controlled study of Shugan jieyu capsule in the treatment of mild and moderate depression. *Chin. J. N. Drugs* 18 413–416.

[B31] Sun Y. Tian G. Shi K. Sun X. Li X. Zeng W. (2018). A comparative study of shugan jieyu capsule and escitalopram oxalate in the treatment of hypertension with anxiety and depression. *Chin. J. Evid. Based Cardiovasc. Med.* 10 1478–1487.

[B32] Thompson G. J. (2018). Neural and metabolic basis of dynamic resting state fMRI. *Neuroimage* 180(Pt B) 448–462. 10.1016/j.neuroimage.2017.09.010 28899744 PMC5844792

[B33] Wang C. Wu H. Chen F. Xu J. Li H. Li H. (2018). Disrupted functional connectivity patterns of the insula subregions in drug-free major depressive disorder. *J. Affect. Disord.* 234 297–304. 10.1016/j.jad.2017.12.033 29587165

[B34] Wang L. Wei Q. Wang C. Xu J. Wang K. Tian Y. (2019). Altered functional connectivity patterns of insular subregions in major depressive disorder after electroconvulsive therapy. *Brain Imaging Behav.* [Epub ahead of print]. 30610527 10.1007/s11682-018-0013-z

[B35] Wang S. H. Zhang Z. J. Guo Y. J. Zhou H. Teng G. J. Chen B. A. (2009). Anhedonia and activity deficits in rats: impact of post-stroke depression. *J. Psychopharmacol.* 23 295–304. 10.1177/0269881108089814 18562439

[B36] Wong C. Lomber S. G. (2019). Stable delay period representations in the posterior parietal cortex facilitate working-memory-guided obstacle negotiation. *Curr. Biol.* 29 70–80.e3. 10.1016/j.cub.2018.11.021 30581021

[B37] Xie H. Calhoun V. D. Gonzalez-Castillo J. Damaraju E. Miller R. Bandettini P. A. (2018). Whole-brain connectivity dynamics reflect both task-specific and individual-specific modulation: a multitask study. *Neuroimage* 180(Pt B) 495–504. 10.1016/j.neuroimage.2017.05.050 28549798 PMC5700856

[B38] Yan M. Xiao S. Hu M. (2016). The Chinese translation, reliability and validity of some depression scales in China. *Chin. J. Ment. Health* 30 501–505.

[B39] Yang X. W. (2015). Curative effect of Shugan jieyu capsule on depression with migraine. *J. Shandong Med. Coll.* 37 105–107.

[B40] Yechiam E. Ben-Eliezer D. Ashby N. J. S. Bar-Shaked M. (2019). The acute effect of *Hypericum perforatum* on short-term memory in healthy adults. *Psychopharmacology (Berl.)* 236 613–623. 10.1007/s00213-018-5088-0 30382352

[B41] Zhen L. Peng G. Zou K. Zou X. (2018). A meta-analysis of the efficacy of shugan jieyu capsule in treating post-stroke depression and improving daily living ability. *Chin. J. Gerontol.* 38 3959–3963.

