# Supplementary material for: Improved Resting-State Functional Dynamics in Post-stroke Depressive Patients After Shugan Jieyu Capsule Treatment
Source: Front Neurosci. 2020 Apr 16;14:297. doi: 10.3389/fnins.2020.00297 (PMC7177051; doi:10.3389/fnins.2020.00297)
Supplement: Supplementary file 1 [file Table_1.DOCX]

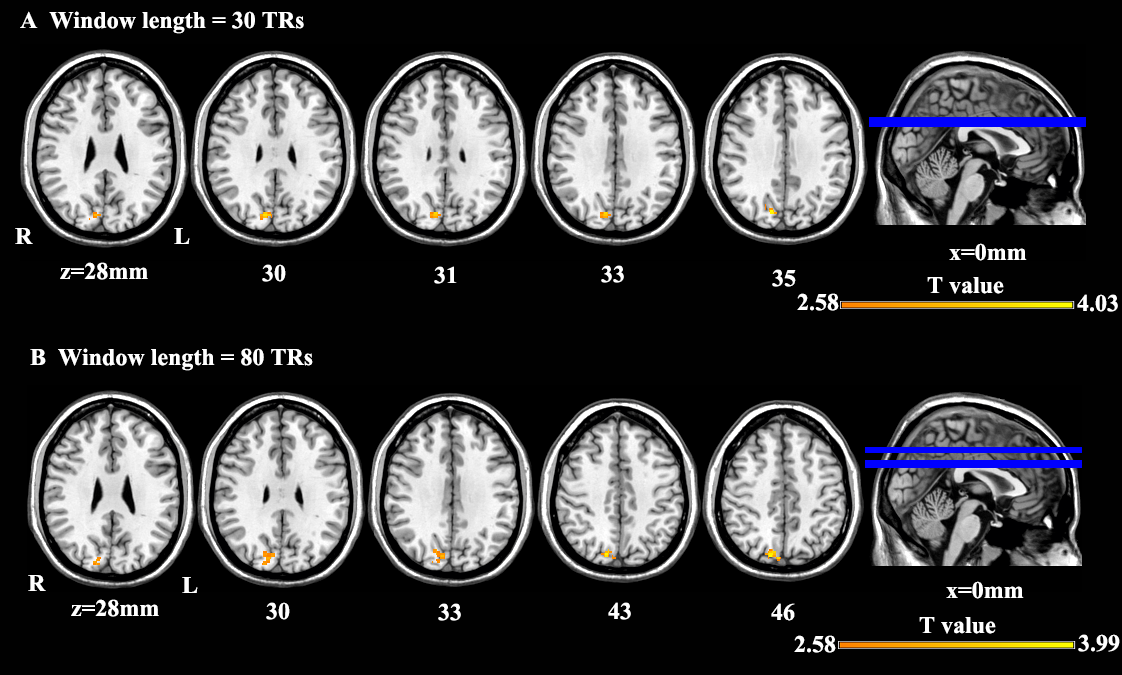


**Supplementary Figure 1. Reproducibility results in different sliding window length.** Two additional sliding-window lengths were chosen with 30 TRs (75s) and 80 TRs (200s). The significant threshold was set to *p* < 0.01 at the voxel level, and GRF correction at the cluster level to *p* < 0.05. Warm color scale indicates regions with increased dALFF value in SG8W, compared to SG0W. Numbers below each axial slice refer to the z-plane coordinates of the MNI space, respectively. L, left; R, right; SG0W, post-stroke depression patients at baseline; SG8W, post-stroke depression patients after 8 weeks.
